# Golgi engineering of CHO cells by targeted integration of glycosyltransferases leads to the expression of novel Asn-linked oligosaccharide structures at secretory glycoproteins

**DOI:** 10.1186/1753-6561-7-S6-P84

**Published:** 2013-12-04

**Authors:** Tobias Reinl, Nicolas Grammel, Sebastian Kandzia, Eckart Grabenhorst, Harald S Conradt

**Affiliations:** 1 Dept. Cell Engineering, Feodor-Lynen-Str. 35, 30625 Hannover, Germany; 2 Dept. Mass Spectrometry, Feodor-Lynen-Str. 35, 30625 Hannover, Germany; 3 Dept. Glycosylation Analysis GlycoThera GmbH, Feodor-Lynen-Str. 35, 30625 Hannover, Germany

## Background and novelty

N-glycans constitute an important information carrier in protein-driven signaling networks. Amongst others, N-glycans contribute to protein folding quality, adjust protein turnover and operate as address label for targeting proteins to specific cells and tissues [1]. Hence, the composition of N-glycans attached to recombinant glycoprotein therapeutics is vital for in-vivo therapeutic efficacy and strongly depends on the choice of the expression host [2,3]. Due to absence or silencing of glycosyltransferase genes homologue to human enzymes, biotechnologically used cell lines are limited by their intrinsic glycosylation machinery and produce host specific glycoforms.

**Figure 1:**
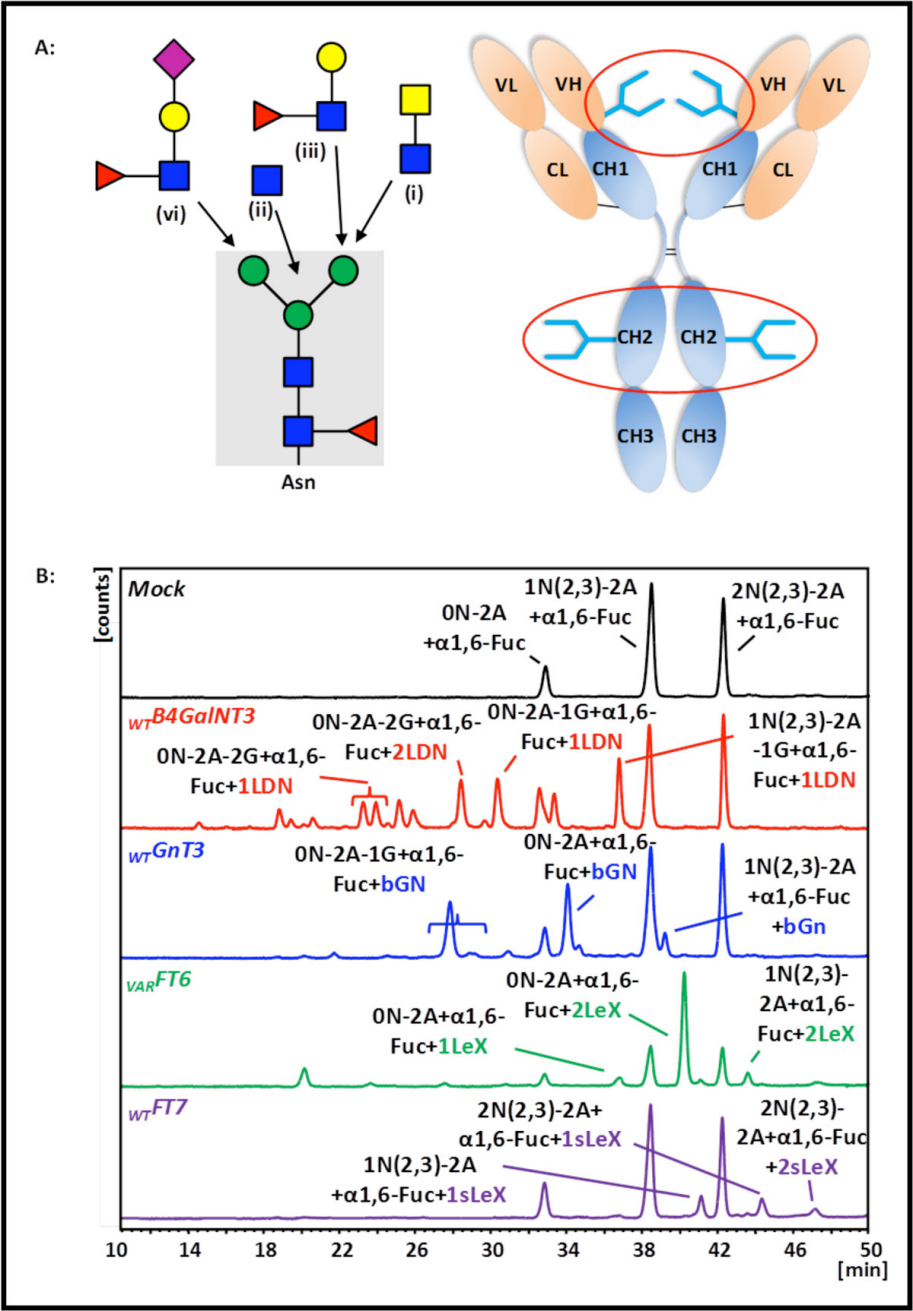
**(A)** Non-reducing terminal oligosaccharide motifs attached to N-glycans of specific human glycoproteins (left side). Scheme of model glycoprotein Cetuximab with CH2- and VH-domain N-glycans (right side). **(B) **NP-HPLC-FLD elution profiles of 2-AB labeled oligosaccharides from VH-domain of Cetuximab after co-expression of the indicated glycosyltransferases.

Cetuximab, a therapeutic chimeric mouse/human monoclonal antibody (IgG1), is N-glycosylated both at the CH2-domain (Asn299) and at the VH-domain (Asn88) (Figure 1A). Sold under the trade name Erbitux^®^, Cetuximab is expressed from a murine myeloma cell line and targets the human EGF receptor [4], which is overexpressed in about 1/3 of all human cancers. The antibody is highly decorated with the αGal-epitope (Galα1-3Galβ1-4GlcNAc) which has been shown to result in fatal allergic/hypersensitivity response in several patients [5].

The design of new quality-optimized and functionally improved biopharmaceuticals with properties conferred by host cell unrelated N-glycans requires a rational Golgi engineering strategy. Here, we apply GET, a system that enables the positioning of a desired catalytic glycosyltransferase activity into a favorable localization within the intracellular glycosylation machinery, to suspension CHO cells developed to secrete suitable amounts (200 μg/ml) of Cetuximab as a model glycoprotein. The presented Golgi engineering project aims in the extension of the intrinsic glycosylation repertoire enabling CHO cells to produce new human-type glycosylation motifs as indicated in Figure 1A: (i) GalNAcβ1,4GlcNAc-R (LacdiNAc, LDN),(ii) GlcNAc in β1,4 linkage to central mannose residue (bisecting GlcNAc, bGN), (iii) Galβ1,4(Fucα1,3)GlcNAc-R (Lewis^X^, Le^X^) and (iv) NeuAcα2,3Galβ1,4 (Fucα1,3)GlcNAc-R (Sialyl-LewisX, sLe^X^). To assemble (ii) and (iv), we co-express GnT3 and FT7. As shown earlier, the latter enzyme catalyzes fucosylation exclusively of (iv). Therefore, we included in our study a variant of FT6 that is targeted to the early Golgi compartment with the aim to additionally generate structure (iii) [6,7]. The uncommon LDN motif (i) which is e.g. detected on lutropin is assembled by human B4GalNT3 [8,9]. We analyze oligosaccharides released from the products of genetically engineered CHO cells based on the resolution of single glycosylation sites of VH- and CH2- glycopeptides by quantitative NP-HPLC-FLD and use our comprehensive oligosaccharide standard library to identify novel oligosaccharide motifs.

## Experimental approach

Cloning of human glycosyltransferases and engineering of _VAR_FT6 [7] as well as construction of pGET expression plasmids encoding either the heavy and light chain of Cetuximab or the glycosyltransferase cDNAs was done acc. to standard DNA technologies. A stable clone with Cetuximab titers of 200 μg/ml and doubling times of 25 hours was selected after transfection of pGET-Cetuximab in CHO cells. This clone was either mock- or co-transfected with pGET plasmids encoding the indicated glycosyltransferases. After shake flask subcultivation for 72 h Cetuximab was purified from supernatants, digested and applied to RP-HPLC peptide mapping. CH2- and VH-domain glycopeptides were separated and oligosaccharides were enzymatically released. After 2-AB labeling, the isolated oligosaccharides were subjected to quantitative NP-HPLC-FLD and ESI-TOF-MS and MS/MS analysis. Oligosaccharide structures were unambiguously identified in comparison to GlycoThera's reference standard oligosaccharide library.

## Results and discussion

**Figure 2:**
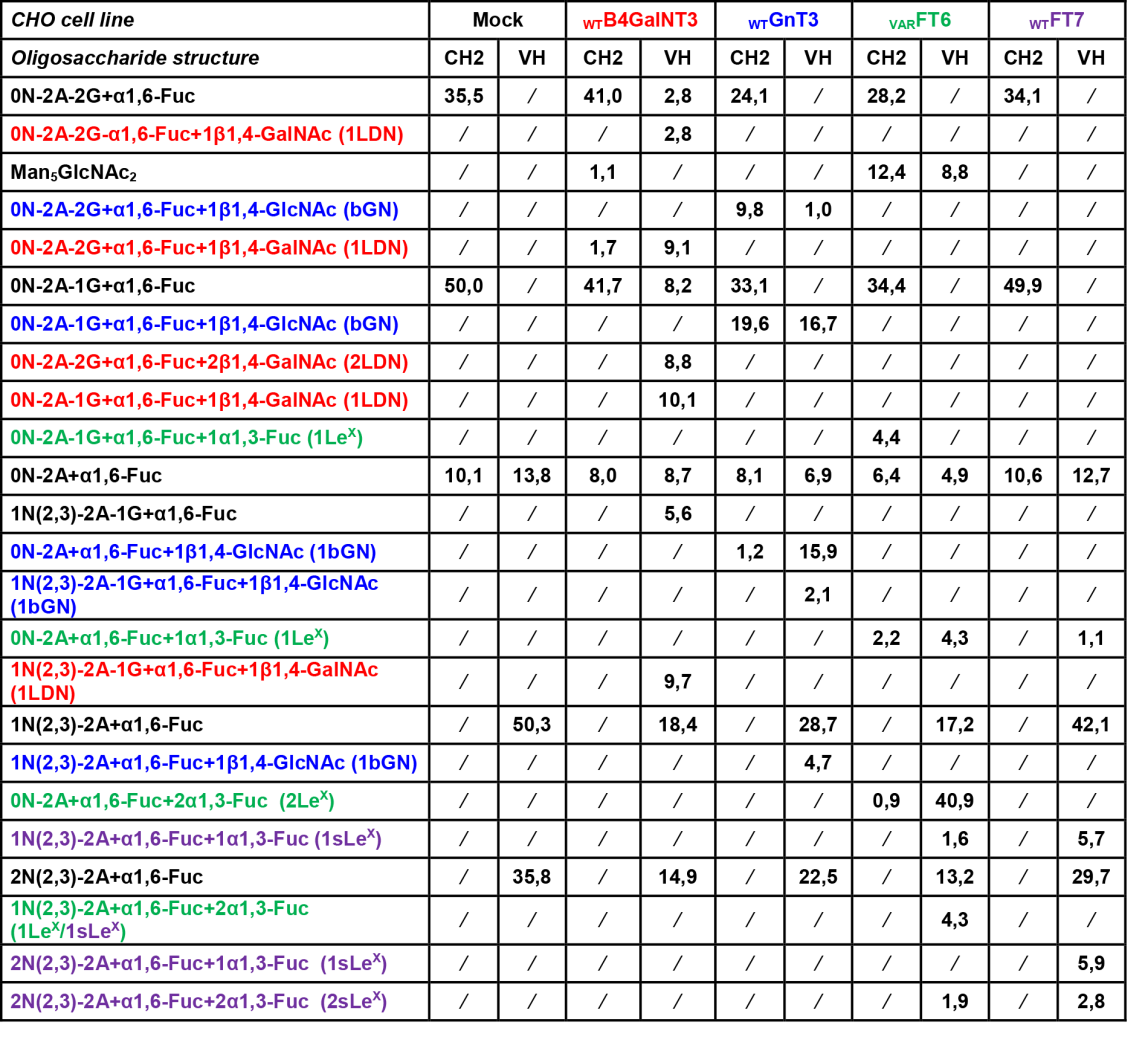
Amount of oligosaccharide structures detected on CH2- and VH-domain of Cetuximab after heterologous glycosyltransferase co-expression (given in% peak area values after integration of NP-HPLC-FLD chromatograms).

In combination with our site specific and quantitative micro glycan structure analysis we provide a modular system (GET) for the customized assembly of novel CHO unrelated oligosaccharide motifs. As exemplified for VH-domain, the NP-HPLC-FLD elution profiles of 2-AB labeled oligosaccharides after heterologous co-expression of Cetuximab and the indicated glycosyltransferases are shown in Figure 1B. Quantitative results of all oligosaccharide structures are given in Figure 2. The Mock-transfected control approach reveals the intrinsic glycosylation repertoire of our stable CHO cell clone. Cetuximab is decorated with agalactosylated (35,5%), mono- (50,0%) and di-galactosylated (10,1%) diantennary complex-type N-glycans containing proximal α1,6-linked fucose at the CH2-domain. VH-domain N-glycans consist of neutral (13,8%), mono- (50,3%) and di-sialylated (35,8%) oligosaccharide structures. Whereas N-glycans from the market product Erbitux^® ^produced in SP2/0 cells are extensively decorated with Galα1,3Gal and NeuGc (data not shown), those allergenic structures are not detected in Cetuximab N-glycans from our CHO cell clone.

The heterologous co-expression of wildtype B4GalNT3, GnT3 and FT7 and genetically modified FT6 results in the formation of the uncommon LacdiNAc motif (ca. 40%), the Lewis^X ^and di-Lewis^X ^structures (ca. 50%) and Sialyl-Lewis^X ^(ca. 15%) almost exclusively on oligosaccharides from the VH-domain. Relevant modification of both VH-domain (ca. 40%) and CH2-domain glycans (ca. 30%) is only achieved by GnT3 catalyzed attachment of bisecting GlcNAc. In addition, glycosyltransferase co-expression leads to charge state reduction of oligosaccharides by depletion of suitable acceptors for endogenous sialyltransferases. The strongest reduction in the content of neuraminic acid at VH-domain was observed by co-expression of _VAR_FT6 (ca. 55% reduction) and _WT_B4GalNT3 (ca. 50% reduction).

As a conclusion, Golgi engineering endows CHO cells to assemble significant amounts of LacdiNAc, bisecting GlcNAc, Lewis^X ^and Sialyl-Lewis^X ^to Cetuximab N-glycans (Figure 1B and Figure 2). Therefore, our glycosylation engineering strategy provides a tool to produce tailored N-glycosylation variants with defined structural motifs. As demonstrated, the tailored addition of bisecting GlcNAc to CH2-domain N-glycans increases ADCC of an αCD20 therapeutic mAB [10]. We therefore assume that the presented structural motifs exhibit novel therapeutic properties (ADCC, CDC, tissue specificity, serum half-life). Our strategy represents a relevant basis for the development of biotherapeutics and biobetters with potentially improved pharmacokinetics, pharmacodynamics, safety properties and in vivo therapeutic efficacy.
